# The value of complete remission according to positron emission tomography prior to autologous stem cell transplantation in lymphoma: a population-based study showing improved outcome

**DOI:** 10.1186/s12885-021-08225-5

**Published:** 2021-05-04

**Authors:** Kristina Noring, Mattias Carlsten, Kristina Sonnevi, Björn Engelbrekt Wahlin

**Affiliations:** 1 Division of Hematology, Department of Medicine, Huddinge, Karolinska Institutet, Stockholm, Sweden; 2 Hematology Unit, Karolinska University Hospital, Solna, Stockholm, Sweden; 3 Center for Hematology and Regenerative Medicine, Department of Medicine, Karolinska Institutet, Huddinge, Stockholm, Sweden

**Keywords:** Lymphoma, B cell, T cell, ASCT, Autologous stem-cell transplantation, PET/CT, Positron emission tomography/computerized tomography, PET

## Abstract

**Background:**

Chimeric antigen-receptor T-cell and bispecific antibody therapies will likely necessitate a reconsideration of the role of autologous stem-cell transplantation (ASCT) in lymphoma. Patients who are likely to profit from ASCT need to be better identified.

**Methods:**

Here, we investigated the value of positron emission tomography/computerized tomography (PET/CT) before ASCT. All 521 patients transplanted for lymphoma 1994–2019 at Karolinska (497 conditioned with BEAM) were included.

**Results:**

Outcome improved over three calendar periods 1994–2004, 2005–2014, 2015–2019 (2-year overall survival [OS]: 66, 73, 83%; *P* = 0.018). Non-relapse mortality (NRM) at 100 days over the three periods were 9.8, 3.9, 2.9%, respectively. The OS improvement between 1994 and 2004 and 2005–2014 was due to lower NRM (*P* = 0.027), but the large OS advance from 2015 was not accompanied by a significant reduction in NRM (*P* = 0.6). The fraction of PET/CT as pre-ASCT assessment also increased over time: 1994–2004, 2%; 2005–2014, 24%; 2015–2019, 60% (*P* < 0.00005). Complete responses (PET/CT-CR) were observed in 77% and metabolically active partial responses (PET/CT-PR) in 23%. PET/CT-CR was a predictor for survival in the entire population (*P* = 0.0003), also in the subpopulations of aggressive B-cell (*P* = 0.004) and peripheral T-cell (*P* = 0.024) lymphomas. Two-year OS and progression-free survival (OS/PFS) for patients in PET/CT-CR were in relapsed/refractory aggressive B-cell lymphoma 87%/75% and peripheral T-cell lymphoma 91%/78%. The corresponding figures in PET/CT-PR were 43%/44 and 33%/33%. Patients with solitary PET/CT-positive lesions showed acceptable outcome with ASCT followed by local irradiation (2-year OS/PFS 80%/60%). CT was less discriminative: 2-year OS/PFS: CT-CR, 76%/66%; CT-PR, 62%/51%. Outcome was inferior after BEAC compared with BEAM conditioning.

**Conclusions:**

We conclude that the improved outcome reflects better, PET/CT-informed, identification of patients who should proceed to ASCT. The excellent survival of patients in PET/CT-CR indicates that ASCT should remain part of standard therapy for lymphoma.

## Background

Even though effective non-chemotherapeutic treatment options for lymphomas, such as chimeric antigen-receptor (CAR)-T cell and bispecific antibody therapies, are rapidly emerging, high-dose chemotherapy followed by autologous stem-cell transplantation (ASCT) remains the standard therapeutic option for many patients. Infections are the most common complication after ASCT and the largest contributor to non-relapse mortality (NRM) [1]. Another concern is the potential risk of developing secondary myeloid malignancies [1]. Careful patient selection prior to ASCT is essential to maximize patient benefit while keeping the rate of complications as low as possible. Additional tools are needed to further improve patient selection.

We have previously investigated stem-cell harvest yields and clinical characteristics which affect NRM, other toxicities, and the development of secondary myeloid disease, to help inform on timing and patient selection [2]. However, another important aspect of patient selection is the strength of the indication, which differ by diagnosis, primary or relapsed/refractory setting, and remission status.

Historically, computerized tomography (CT) has been used to assess patients’ remission status prior to ASCT. The combination of 18-fluorodeoxyglucose positron emission tomography and CT (PET/CT) enables radiologic mapping of metabolic activity and can be used for identifying and staging the lymphoid malignancies which have elevated glucose consumption. PET/CT is superior to CT in discriminating between malignant and non-malignant residual tissue, decreasing the risk of incorrectly identifying radiologic remnants post-chemotherapy as active lymphoma [3]. The utility of PET/CT before ASCT to predict outcome has been shown in several lymphoma entities [4–16], although negative series also exist [17, 18]. In these studies, several different conditioning regimens were used, and the timing of the PET/CT differed. We wanted to investigate whether outcome after ASCT had improved with the increasing use of pre-transplant PET/CT and to assess outcome in 521 consecutive lymphoma patients treated with ASCT (497 conditioned with BEAM) in a population-based single-center study.

## Methods

All lymphoma patients who underwent ASCT at the Hematology Unit, Karolinska University Hospital, from 1 January 1994 until 31 October 2019 were included in this retrospective single-center observational study (excluding patients with primary CNS lymphoma). The material is population-based, since the Hematology Unit is the only site conducting this procedure in the Stockholm-Gotland Healthcare Region (population, 2.4 million). Patient data were extracted from electronic medical files. BEAM was dosed day − 7: BCNU 300 mg/m^2^ 4-h infusion, intrathecal methotrexate 12 mg; days − 6, − 5, − 4, − 3: etoposide 200 mg/m^2^ 2-h infusion, cytarabine 400 mg/m^2^ 12-h infusion (day − 3 also intrathecal methotrexate 12 mg); day − 2: melphalan 140 mg/m^2^ (intrathecal methotrexate was omitted in patients with indolent, T-cell and Hodgkin lymphoma). BEAC was dosed day − 6: BCNU 300 mg/m^2^ 4-h infusion; days − 5, − 4, − 3, − 2: etoposide 100 mg/m^2^ twice daily, cytarabine 100 mg/m^2^ 0.5-h infusion twice daily, cyclophosphamide 35 mg/kg once and mesna 14 mg/kg four times daily. BCNU-thiotepa was dosed BCNU 400 mg/m^2^ 1-h infusion day − 6; thiotepa 5 mg/kg twice daily days − 5 and − 4. Investigations of stem-cell harvests (median, 6 million CD34+ cells per kg), NRM, and procedure-related toxicities have been published before [2]. Refractory disease was defined as a previous failure of chemotherapy or relapse within 3 months of treatment.

### CT and PET/CT

Complete and partial response (CR; PR) were defined as responses with at least 50% reduction of lymphoma size and separated by the absence or presence of a lymph node or focal lesion in any organ ≥1 cm, or as specified by the reviewing radiologist. PET/CT responses were defined according to the Lugano classification (PET/CT-CR was Deauville score ≤ 3) [3]. PET/CT-PR denotes a shrinking of lymphoma mass but still metabolically active disease.

### Statistical analysis

Patients were followed from ASCT until death or last follow-up (November 2019). Depending on the nature of the independent variables, relationships between them were investigated using the χ^2^, Wilcoxon or Spearman test. Overall, lymphoma-specific, and progression-free survivals (OS; LSS; PFS) were calculated from the day of ASCT until the day of death (OS), death from lymphoma (LSS), death or progression of disease (PFS). Univariate and multivariate analyses were conducted using Kaplan-Meier curves and Cox regression; the proportional hazards assumption was checked with graphs based on Schoenfeld residuals. All *P* values are two-tailed and calculated using Stata 14.2 (StataCorp, College Station, TX, USA). *P* < 0.05 was considered significant.

This study was approved by the Ethics Committee, Stockholm (Ref no. 2012/783–31/3 with Amendments 2015/327–32 and 2016/2379–32).

## Results

**Fig. 1:**
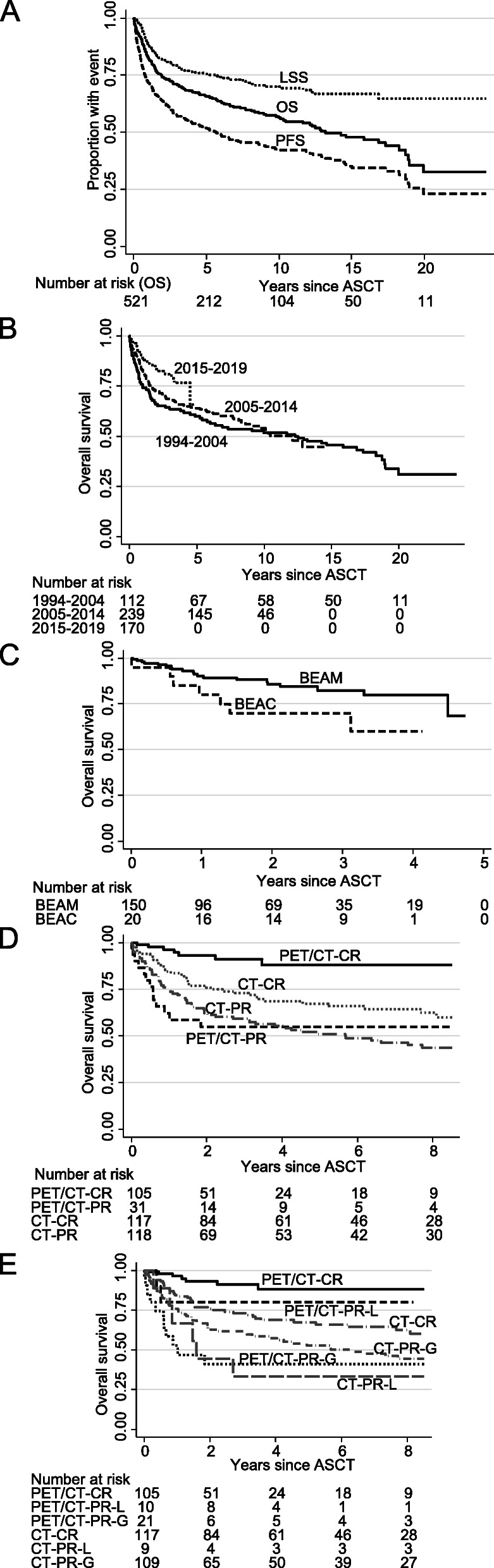
Kaplan-Meier graphs of (**a**) overall, lymphoma-specific, and progression-free survival in all patients (OS; LSS; PFS), **b** OS by calendar period, **c** BEAM or BEAC conditioning in the last calendar period, **d** partial or complete remission (PR; CR) status according to computerized tomography (CT) or 18-flourodeoxyglucose positron emission tomography (PET)/CT, **e** PR or CR status according to CT and PET/CT with PR patients locally treated after ASCT (PR-L) as a separate group, compared with generalized PR (PR-G); the last two graphs are cut at 8 years due to the different observation times between CT and PET/CT patients. Abbreviation: ASCT, autologous stem-cell transplantation

Five hundred twenty-one lymphoma patients underwent ASCT between 1994 and 2019 (Table 1). Most patients were male (63%) and the median age was 57 years (range, 18–72). At last follow-up (November 2019), the median follow-up time in survivors was 5.3 years (range, 0.1–24.3). The median OS, LSS, and PFS were 13.2, not reached, and 5.7 years (Fig. 1a). OS and PFS were 74 and 66% at 2 years and 64 and 52% at 5 years (Table 2). The conditioning regimen was BEAM in 497 patients. Because of occasional melphalan shortages from 2015 onwards, BEAC was used in 20 patients. Three patients with CNS relapse of systemic lymphoma were conditioned with BCNU-thiotepa and the first patient in 1994 received cyclophosphamide-total body irradiation. There was improved outcome over the three calendar periods 1994–2004, 2005–2014, 2015–2019 (2-year OS: 66, 73, 83%; *P* = 0.018; Fig. 1b), partly explained by decreasing NRM (at 100 days, 9.8, 3.9, 2.9%, respectively; previously thoroughly described [2]). The OS improvement between 1994 and 2004 and 2005–2014 was due to lower NRM (*P* = 0.027), but the 10-percentage point OS advance between 2005 and 2014 and 2015–2019 (*P* = 0.029) was not accompanied by a significant reduction in NRM (*P* = 0.6). Rituximab use increased in B-cell disease (30, 96, 100% over the three calendar periods; *P* < 0.00005) but could not explain the better outcome (Table 1). Neither was it caused by changes in conditioning because the BEAM regimen never changed, and, in the last calendar period, there was a clear tendency for inferior outcome in patients treated with BEAC compared with BEAM (Fig. 1c), with *P* = 0.059 for OS, *P* = 0.034 for LSS, and *P* = 0.062 for PFS. When BEAC patients were excluded, not only OS and NRM, but also LSS was significantly better in the last calendar period (*P* = 0.040), suggesting the emergence of a new lymphoma-specific survival factor.

**Table 1:** Clinical characteristics

	N	per cent	OS	LSS	PFS
			HR (95% CI)	HR (95% CI)	HR (95% CI)
Sex					
Female	193	37%	1	1	1
Male	328	63%	1.12 (0.83–1.50)	1.06 (0.72–1.54)	1.01 (0.79–1.30)
Age, years					
Median (range) 57 (18–72)					
18–45	126	24%	1	1	1
46–55	114	22%	2.04 (1.26–3.31)	2.08 (1.09–3.94)	1.70 (1.15–2.51)
56–65	189	36%	2.99 (1.94–4.61)	2.87 (1.62–5.08)	2.09 (1.47–2.93)
66–72	92	18%	3.26 (1.97–5.40)	3.43 (1.81–6.52)	2.32 (1.54–3.50)
Lymphoma entity					
Aggressive B cell	121	23%	1	1	1
Transformed B cell	107	21%	0.80 (0.53–1.20)	0.66 (0.40–1.09)	1.02 (0.72–1.46)
Indolent B cell	49	9%	0.88 (0.56–1.40)	0.59 (0.32–1.10)	1.15 (0.76–1.74)
Mantle cell	99	19%	0.50 (0.32–0.78)	0.38 (0.21–0.66)	0.65 (0.45–0.95)
T cell	85	16%	0.71 (0.47–1.10)	0.51 (0.29–0.90)	0.80 (0.55–1.18)
Classical Hodgkin	60	12%	0.37 (0.21–0.66)	0.19 (0.08–0.49)	0.54 (0.33–0.87)
Indication for ASCT					
Upfront	183	35%	1	1	1
Relapsed disease	249	48%	1.85 (1.31–2.62)	1.82 (1.17–2.84)	1.50 (1.13–2.00)
Refractory disease	88	17%	2.48 (1.64–3.73)	2.58 (1.53–4.36)	1.95 (1.37–2.77)
Calendar period					
1994–2004	112	21%	1	1	1
2005–2014	239	46%	0.93 (0.67–1.29)	1.15 (0.74–1.77)	0.92 (0.58–1.23)
2015–2019	170	33%	0.54 (0.34–0.87)	0.74 (0.41–1.33)	0.82 (0.57–1.19)
Number of prior lines of chemotherapy					
1	201	41%	1	1	1
2	256	52%	1.65 (1.19–2.29)	1.80 (1.18–2.76)	1.44 (1.09–1.89)
3 or more	37	7%	2.32 (1.33–4.07)	2.70 (1.36–5.39)	1.81 (1.11–2.96)
Conditioning regimen					
BEAM	497	95%	1	1	1
BEAC	20	4%	1.22 (0.57–2.61)	1.57 (0.69–3.59)	1.59 (0.88–2.84)
BCNU-Thiotepa	3	1%	NA	NA	NA
Cy-TBI	1	0%	NA	NA	NA
Rituximab prior to ASCT in B-cell disease					
No	63	17%	1	1	1
Yes	313	83%	0.61 (0.42–0.87)	0.76 (0.47–1.22)	0.73 (0.53–1.02)

*Abbreviations*: *OS* overall survival, *LSS* lymphoma-specific survival, *PFS* progression-free survival, *ASCT* autologous stem-cell transplantation, *NA* not analyzable

**Table 2:** Lymphoma entities

	N	OS				LSS				PFS			
		2 y	5y	10y	P	2y	5y	10y	P	2y	5y	10y	P
All lymphomas	521	74%	66%	56%		82%	76%	70%		64%	52%	42%	
Primary aggressive B-cell lymphomas	121	59%	53%	47%		67%	64%	56%		52%	45%	41%	
DLBCL or high-grade B-cell lymphoma	105	59%	52%	44%		68%	64%	55%		50%	42%	38%	
Not-DLBCL or high-grade B-cell lymphoma	16	60%	60%	60%	0.28	64%	64%	64%	0.55	61%	61%	61%	0.14
FL grade 3B	2												
Burkitt lymphoma	3												
PTLD	2												
PMBCL or greyzone lymphoma	3												
Plasmablastic lymphoma	5												
Lymphomatoid granulomatosis grade III	1												
Upfront patients	7	86%	86%	86%		100%	100%	100%		86%	86%	86%	
Relapsed/refractory patients	114	58%	51%	44%	0.19	65%	62%	53%	0.070	50%	43%	38%	0.09
Transformed B-cell lymphomas	107	71%	63%	52%		78%	69%	64%		59%	42%	35%	
Transformed FL	72	76%	71%	56%		81%	75%	68%		65%	48%	38%	
Transformed not-FL	35	61%	41%	41%	0.22	73%	49%	49%	0.35	48%	30%	30%	0.28
Transformed MZL	18												
Transformed CLL/SLL (Richter)	5												
Transformed WM	3												
Transformed NLPHL	2												
Transformed indolent B-cell lymphoma	7												
Upfront patients	28	92%	92%	92%		95%	95%	95		68%	55%	41%	
Relapsed/refractory patients	79	64%	54%	41%	0.006	73%	61%	55%	0.009	55%	38%	33%	0.13
Primary and transformed aggressive B-cell lymphomas	228	64%	58%	50%		72%	67%	60%		55%	44%	39%	
Upfront patients	35	90%	90%	90%		96%	96%	96%		72%	63%	54%	
Relapsed/refractory patients	193	60%	53%	44%	0.002	68%	63%	54%	0.001	52%	42%	37%	0.028
Indolent B-cell lymphomas	49	80%	59%	44%		90%	75%	65%		60%	45%	26%	
FL grade 1-3A	39	78%	57%	43%		88%	76%	69%		60%	44%	31%	
Not-FL grade 1-3A	10	89%	63%	51%	0.55	100%	71%	57%	0.24	57%	46%	11%	0.48
MZL	1												
CLL/SLL	6												
WM	1												
NLPHL	2												
All indolent lymphomas	156	74%	61%	48%		82%	71%	64%		59%	43%	31%	
Not transformed	49	80%	59%	44%		90%	75%	65%		60%	45%	26%	
Transformed	107	71%	63%	52%	0.77	78%	69%	64%	0.65	59%	42%	35%	0.77
All follicular lymphomas	111	77%	65%	50%		83%	75%	68%		63%	47%	35%	
Not transformed	39	78%	57%	43%		88%	76%	69%		60%	44%	31%	
Transformed	72	76%	71%	56%	0.58	81%	75%	68%	0.63	65%	48%	38%	0.69
Mantle cell lymphoma	99	86%	75%	73%		91%	82%	82%		80%	62%	43%	
Classical	83	90%	78%	78%		95%	86%	86%		84%	67%	47%	
Non-classical	16	66%	58%	43%	0.021	70%	62%	62%	0.025	61%	37%	18%	0.005
Blastic	9												
Pleomorphic or P53+ or Ki67 > 60%	7												
Upfront patients	87	90%	78%	78%		93%	84%	84%		83%	66%	45%	
Relapsed/refractory patients	12	58%	49%	32%	0.006	81%	67%	67%	0.30	58%	40%	40%	0.054
T-cell lymphomas	85	74%	69%	53%		83%	79%	71%		65%	54%	45%	
PTCL	78	79%	73%	56%		87%	83%	74%		70%	58%	48%	
Non-PTCL	7	18%	18%	ND	0.004	25%	25%	ND	0.013	0%	0%	0%	0.002
Sézary/ATLL	3												
ENKTCL	4												
PTCL, ALCL	25	79%	79%	74%		91%	91%	91%		80%	75%	70%	
PTCL, non-ALCL	53	78%	69%	41%	0.08	85%	78%	63%	0.09	65%	47%	33%	0.023
AITL/FHTCL	29												
PTCL-NOS	17												
EATCL/HSTCL/SPTCL/Lennert	7												
Upfront PTCL patients	56	81%	75%	67%		86%	80%	71%		70%	60%	55%	
Relapsed/refractory PTCL patients	22	73%	68%	39%	0.07	89%	89%	80%	0.44	68%	54%	38%	0.21
Classical Hodgkin lymphoma	60	85%	83%	74%		92%	90%	90%		71%	66%	60%	

*Abbreviations*: *OS* overall survival, *LSS* lymphoma-specific survival, *PFS* progression-free survival, *DLBCL* diffuse large B-cell lymphoma, *FL* follicular lymphoma, *PTLD* post-transplantation lymphoproliferative disorder, *PMBCL* primary mediastinal B-cell lymphoma, *MZL* marginal zone lymphoma, *CLL* chronic lymphocytic leukemia, *SLL* small lymphocytic lymphoma, *WM* Waldenström macroglobulinemia, *NLPHL* nodular lymphocyte-predominant Hodgkin lymphoma, *PTCL* peripheral T-cell lymphoma, *ALCL* anaplastic large cell lymphoma, *AITL* angioimmunoblastic T-cell lymphoma, *FHTCL* follicular helper T-cell lymphoma, *EATCL* enteropathy-associated T-cell lymphoma, *HSTCL* hepatosplenic T-cell lymphoma, *SPTCL* subcutaneous panniculitis-like T-cell lymphoma, *ATLL* adult T-cell leukemia/lymphoma, *ENKTCL* extranodal NK/T-cell lymphoma, nasal type

### The emergence of PET/CT

We compared the prognostic value of pre-transplant CT and PET/CT. Cases were excluded if the last radiology evaluation prior to ASCT was PR but conducted interim (before the last course of chemotherapy prior to conditioning). All assessments showing CR in the last evaluation were included, either when conducted interim or directly before the ASCT. Thus, the last evaluation of response prior to ASCT was CT in 235 patients (62%) and PET/CT in 136 (36%), the remaining 8 (2%) were assessments using MRI, bone marrow biopsy, or palpation. Over time, PET/CT became increasingly common: PET/CT constituted 2% and CT 98% of the final evaluations 1994–2004, the corresponding numbers were 24 and 75% 2005–2014, and 60 and 37% 2015–2019 (*P* < 0.0005). Of patients evaluated with PET/CT prior to ASCT, 77% were in CR and 23% in PR (Table 3).

**Table 3:** Remission status by PET/CT and CT

	N	percent	OS			LSS			PFS		
			2y	5y	P	2y	5y	P	2y	5y	P
PET/CT	136	100%									
PET/CT-CR	105	77%	93%	88%		94%	92%		81%	67%	
PET/CT-PR	31	23%	55%	55%	0.0003	68%	68%	0.0009	45%	41%	0.004
PET/CT-PR-local	10	7%	80%	80%		90%	90%		60%	50%	
PET/CT-PR-general	21	15%	41%	41%	< 0.00005	56%	56%	0.0002	36%	36%	0.003
Aggressive B-cell lymphoma	72										
PET/CT-CR	50	69%	90%	76%		90%	85%		73%	55%	
PET/CT-PR	22	31%	50%	50%	0.004	59%	59%	0.006	45%	39%	0.032
Relapsed/refractory	54										
PET/CT-CR	36	67%	87%	64%		87%	80%		75%	48%	
PET/CT-PR	18	33%	43%	43%	0.013	50%	50%	0.008	44%	44%	0.15
Peripheral T-cell lymphoma	18										
PET/CT-CR	15	83%	91%	91%		91%	91%		78%	62%	
PET/CT-PR	3	17%	33%	NA	0.024	50%	NA	0.19	33%	NA	0.27
Classical Hodgkin lymphoma	25										
PET/CT-CR	21	84%	100%	100%		100%	100%		89%	67%	
PET/CT-PR	4	16%	75%	75%	0.083	100%	100%	1	50%	50%	0.37
CT	235										
CT-CR	117	50%	76%	67%		83%	78%		66%	55%	
CT-PR	118	50%	62%	51%	0.035	70%	62%	0.022	51%	36%	0.004
Aggressive B-cell lymphoma	86										
CT-CR	34	40%	59%	52%		69%	69%		50%	35%	
CT-PR	52	60%	52%	47%	0.81	57%	52%	0.25	46%	37%	0.85
Relapsed/refractory	77										
CT-CR	30	39%	50%	45%		64%	64%		46%	30%	
CT-PR	47	61%	49%	44%	0.99	54%	49%	0.38	43%	35%	0.99
Peripheral T-cell lymphoma	44										
CT-CR	27	61%	74%	63%		77%	71%		67%	56%	
CT-PR	17	39%	76%	76%	0.93	92%	92%	0.22	65%	44%	0.25
Classical Hodgkin lymphoma	13										
CT-CR	3	23%	100%	100%		100%	100%		67%	67%	
CT-PR	10	77%	70%	70%	0.21	70%	70%	0.32	50%	50%	0.34

*Abbreviations*: *PET/CT* positron emission tomography/computerized tomography, *CT* computerized tomography, *CR* complete remission, *PR* partial remission, *NA* not analyzable

Both with CT and PET/CT, PR compared with CR was a poor prognostic marker (OS, *P* = 0.035, *P* = 0.0003, respectively). However, PET/CT was markedly more powerful than CT (Table 3; Fig. 1d). For example, at 2 years, the OS rates were in CT-CR 76% and CT-PR 62%, and in PET/CT-CR 93% and PET/CT-PR 55% (Table 3). Because CT had been conducted more in the earlier times with higher NRM, the analysis was repeated with restriction to the last calendar period (2015–2019) with similar results (2-year OS: CT-CR 81%, CT-PR 70%, PET/CT-CR 96%, PET/CT-PR 63%). At the start of conditioning, in patients with PET/CT-CR, 68% had normal C-reactive protein (< 3 mg/L), 25% 3–10 mg/L, and 7% ≥10 mg/L, whereas in PET/CT-PR, the corresponding numbers were 41, 38, and 21% (*P* = 0.005). There were no C-reactive protein differences between CT-CR and CT-PR (*P* = 0.65).

We further investigated the patients transplanted in PR: 19 were given a planned local therapy after ASCT (18, irradiation; 1, splenectomy). That approach appeared useful in the 10 patients where the local therapy was PET-guided (2-year OS/PFS 80%/60%) but not in those 9 where CT defined PR (2-year OS/PFS 44%/44%; Fig. 1e). The PET/CT-PR patients given local therapy (PET/CT-PR-local) was an intermediate prognostic group between PET/CT-CR and PET/CT-PR-general (several FDG-avid sites; Table 3). With respect to OS and PFS, PET/CT-PR compared with PET/CT-CR showed inferior outcome with hazard ratios (HR) 4.0 (95% confidence interval [CI], 1.8–9.1) and 2.4 (95% CI, 1.3–4.6). For comparison, CT-PR compared with CT-CR had HR 1.5 (95% CI, 1.0–2.2) for OS and HR 1.6 (95% CI, 1.2–2.3) for PFS.

### Primary and transformed aggressive B-cell lymphomas

**Fig. 2:**
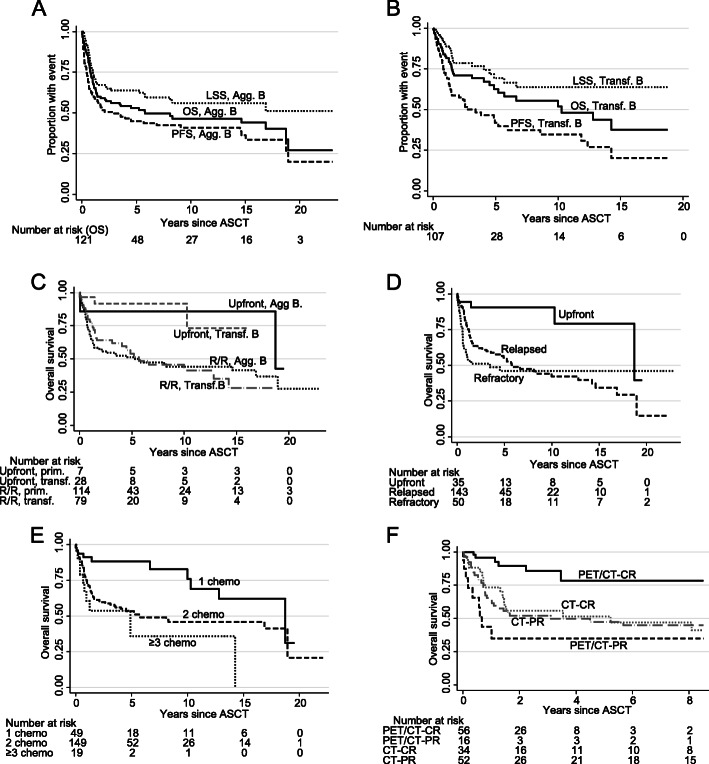
Kaplan-Meier graphs of overall, lymphoma-specific, and progression-free survival (OS; LSS; PFS) in (**a**) primary aggressive (Agg. B) and (**b**) transformed indolent (Transf. B) B-cell lymphoma, **c** OS by upfront and relapsed/refractory status in primary aggressive and transformed indolent B-cell lymphoma, **d** OS in primary aggressive or transformed indolent B-cell lymphoma patients by upfront, relapsed, or refractory status, **e** OS by number of preceding lines of chemotherapy, **f** OS by PR or CR status according to CT and PET/CT (with PET/CT-PR patients locally treated after ASCT grouped with PET/CT-CR); the last graph is cut at 8 years due to the different observation times between CT and PET/CT patients. Abbreviation: ASCT, autologous stem-cell transplantation

In total, 228 (121 primary and 107 transformed) patients were transplanted for aggressive B-cell lymphoma. Outcome did not differ between diffuse large B-cell or other types of primary aggressive lymphoma; likewise, there were no differences between transformed follicular or other transformed indolent lymphomas, nor, overall, between primary or transformed aggressive lymphomas (Table 2), and the curves were roughly similar (Fig. 2a, b). There were no outcome differences between primary or transformed aggressive lymphoma in the upfront or relapsed/refractory setting (Fig. 2c), why the 228 aggressive B-cell patients were subsequently grouped for statistical power. Patients transplanted upfront in first remission had better outcome than those who had had relapsed or refractory disease (Table 2; Fig. 2d), similarly, the number of prior lines of chemotherapy was highly predictive for outcome (OS, *P* = 0.001; LSS, *P* = 0.0002; PFS, *P* = 0.0002; Fig. 2e). PET/CT was a valuable predictive tool for OS (*P* = 0.004), LSS (*P* = 0.006), and PFS (*P* = 0.032), while remission status by CT had no value (Fig. 2f; Table 3).

#### Upfront transplanted primary or transformed aggressive B-cell lymphoma

As shown in Table 2, 35 patients were transplanted upfront, with good OS and PFS (at 2 years 90 and 72%). The small number of events made analysis of the predictive value of PET/CT difficult, but there was a trend for inferior outcome (OS, *P* = 0.061; PFS, *P* = 0.078) in patients with PET/CT-PR. CR or PR according to CT had no prognostic value (*P* ≥ 0.4).

#### Relapsed/refractory transplanted primary or transformed aggressive B-cell lymphoma

There were 193 patients transplanted with relapsed or refractory disease: OS and PFS at 2 years were 60 and 52% (Table 2). The 2-year OS and PFS in patients in PET/CT-CR prior to ASCT were 87 and 75%, but in patients with PET/CT-PR 43 and 44% (*P* = 0.013 and *P* = 0.15); with CT-CR, the 2-year OS and PFS were 50 and 46% and with CT-PR, 49 and 43% (*P* = 0.99 and *P* = 0.99; Table 3). Thus, PET/CT exhibited much stronger predictive value than CT.

### Indolent B-cell lymphomas

**Fig. 3:**
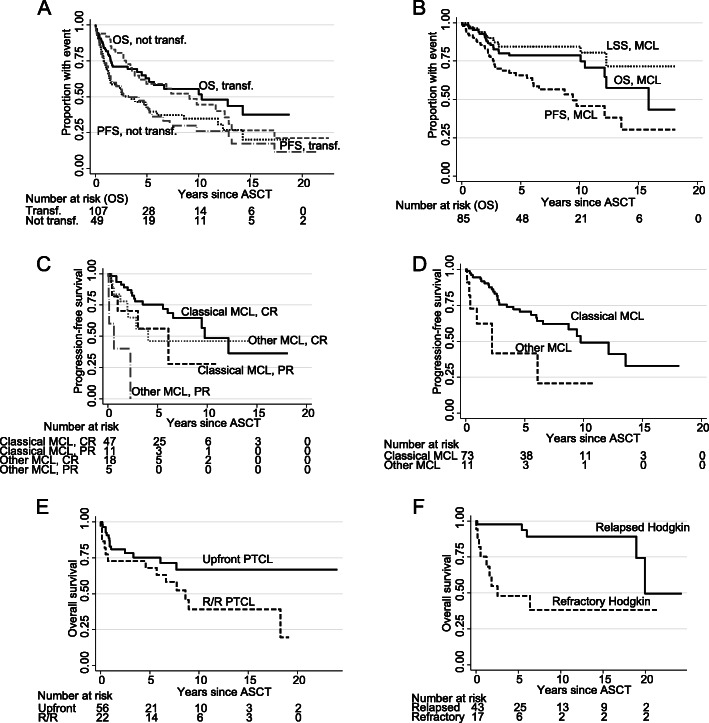
Kaplan-Meier graphs of (**a**) overall and progression-free survival (OS; PFS) in not-transformed (not transf.) and transformed (transf.) indolent B-cell lymphoma, **b** OS, lymphoma-specific survival (LSS), and PFS in mantle cell lymphoma (MCL), **c** PFS by remission status in classical and other types of MCL, **d** PFS in classical and other types of MCL transplanted upfront, **e** OS in peripheral T-cell lymphoma (PTCL) by upfront or relapsed/refractory (R/R) status, **f** OS in Hodgkin lymphoma by relapsed or refractory status. Abbreviation: ASCT, autologous stem-cell transplantation

Short- and long-term outcome of the 49 patients with indolent lymphoma are shown in Table 2; late relapses were seen. In follicular lymphoma, 31% were alive and in remission after 10 years. Outcome was almost identical (Fig. 3a; Table 2) in transformed and not-transformed indolent lymphoma, apart from an expected initial higher mortality in transformed disease. There were too few events for investigating the PET/CT method in not-transformed indolent lymphoma. When combining transformed and not-transformed disease, PET/CT was highly predictive for OS (*P* = 0.0003) and PFS (*P* = 0.013), while CT was not predictive (*P* > 0.5 for both comparisons).

### Mantle cell lymphoma

Eighty-five out of 99 mantle cell lymphoma patients were transplanted upfront in or according to the Nordic MCL2 trial [19]. These patients showed excellent outcome with median PFS 9.4 years (Fig. 3b). Patients with more aggressive variants of MCL (blastic, pleomorphic, TP53+, or Ki67 > 60%) showed poor outcome (Table 2), also after upfront ASCT according to the MCL2 protocol (Fig. 3c). CR compared with PR, regardless whether informed from PET/CT or CT, was highly predictive of outcome (OS, *P* = 0.039; LSS, *P* = 0.044; PFS = 0.025; Fig. 3d). Also in the subset of upfront transplanted patients, CR according to PET/CT or CT predicted outcome (OS, *P* = 0.042; LSS, *P* = 0.049; PFS, *P* = 0.030). Late relapses were seen.

### T-cell lymphoma

Seventy eight out of 85 patients had peripheral T-cell lymphoma, and 56/78 were transplanted upfront in or according to the Nordic NLG-T-01 trial [20]; these patients showed good outcome (Fig. 3e) with 5-year OS at 75% (Table 2). The two-year OS/PFS in patients with peripheral T-cell lymphoma were 91%/78% if PET/CT-CR, 33%/33% if PET/CT-PR (Table 3). In contrast, CT could not predict outcome. Also in upfront transplanted peripheral T-cell lymphoma patients, PET/CT predicted OS (*P* = 0.005) and PFS (*P* = 0.045) but CT did not (*P* ≥ 0.5 for both comparisons).

### Classical Hodgkin lymphoma

These 60 patients, all relapsed/refractory, showed excellent outcome (OS at 5 years, 83%; Table 2). However, refractory disease portended poorer survival than relapsed disease (Fig. 3f; OS, *P* = 0.002; LSS, *P* = 0.022; PFS, *P* = 0.021). In PET/CT-CR, 2-year OS/PFS were 100%/89% and in PET/CT-PR 75%/50%, but there were too few events to achieve statistical significance (Table 3).

### Relapse after ASCT

The estimated fraction of relapses at 2 years were with PET/CT-CR 18%, PET/CT-PR 41%, CT-CR 29%, CT-PR 43%. Two hundred five out of 521 patients relapsed after ASCT, at a median time of 308 days. Of these 205 patients 118 died from lymphoma, 3 from treatment toxicity, 18 from other causes, while 66 stayed alive. Relapse was most common in indolent (53%), transformed (42%), and primary aggressive (44%) B-cell lymphoma. It was seen in 36, 35, and 25% of mantle cell, T-cell, and Hodgkin lymphoma. Five-year OS after relapse was 30%, and particularly poor in aggressive B-cell lymphoma (16%), but 51% in Hodgkin lymphoma. The 44 patients who underwent allogeneic transplantation for relapsed disease showed better survival than those who did not (5-year OS 63% vs 21%; *P* < 0.00005), particularly in aggressive or transformed B-cell lymphoma (5-year OS 70% vs 11%; *P* < 0.00005). No patient had undergone CAR-T cell therapy at last follow-up. Long-term adverse events in our patients, including myeloid disease, have been described previously [2].

## Discussion

This population-based single-center study shows that outcome after ASCT has improved with increasing PET/CT use. PET/CT may verify metabolic remission, identifying patients with truly chemosensitive disease, who have excellent outcome with ASCT. Hence, BEAM followed by ASCT remains an effective treatment option in lymphoma.

Two-year OS and PFS in relapsed/refractory primary or transformed aggressive B-cell lymphoma were 60 and 52%. These figures are not inferior to those reported from the large CAR-T cell trials. In the axicabtagene ciloleucel trial, PFS was 42% at a median follow-up of 15.4 months [21]. In the tisagenlecleucel trial, the intention-to-treat analysis showed OS to be 40% at 1 year, and, in the patients who did proceed to receive CAR-T cell therapy, PFS was about 35% at 18 months [22]. Lastly, the lisocabtagene maraleucel trial showed a 1-year PFS of 44% [23]. Granted, these two modalities are not directly comparable, because only responders to chemotherapy proceed to ASCT and the follow-up times differ; on the other hand, many patients were excluded from therapy in the CAR-T cell trials because of disease progression between leukapheresis and CAR-T cell infusion, and the CAR-T numbers come from clinical trials, not real-world practice, although emerging real-world CAR-T data appear to be equally good [24]. Furthermore, CAR-T cells are vastly more useful than ASCT for patients with stable disease after salvage induction therapy, and, of course, for those who relapse after ASCT. However, with PET/CT, one may identify those patients with aggressive B-cell lymphomas who will benefit from ASCT consolidation: in relapsed/refractory patients with PET/CT-CR, 2-year OS/PFS was 87%/75%. This excellent outcome for PET-negative relapsed/refractory patients is not inferior to the outcome of patients who attained complete remission in the CAR-T cell trials (PFS between 65 and 85% at 1 year) [21–23]—again the comparison limps somewhat because of differences in remission status and patient selection (particularly chemosensitivity) prior to the respective procedure. In our material, it also appears that the small proportion of patients who only have one site of PET positivity might be cured by with ASCT followed by local irradiation or surgery conducted after the bone-marrow regeneration. Involved-field radiotherapy to PET-positive lesions prior to ASCT has been shown to be a successful approach [25]. In PET/CT-CR patients with peripheral T-cell lymphoma, 2-year OS/PFS after ASCT was 91%/78%. These figures compare favourably with other series, including one in which some of our patients participated [20, 26, 27]. Outcome in PET/CT-CR patients with Hodgkin lymphoma was excellent: 2-year OS/PFS 100%/89%.

In mantle cell lymphoma and indolent lymphoma, OS and PFS after ASCT were similar to previous reports, and ASCT remains part of standard therapy [19, 28]. However, late relapses do occur after ASCT; the 10-year PFS was in follicular lymphoma 31% and in mantle cell lymphoma 43%. We believe that CAR-T cells [29, 30] and bispecific antibodies [31, 32] might improve long-term outcome for these slow-growing entities.

In our patients, survival after ASCT improved from 2015, without any improvement of NRM, thus this is probably due to a better identification of patients who should and should not proceed to ASCT. Karolinska’s standard today is upfront ASCT in first remission for patients with transformed indolent B-cell, mantle cell, and peripheral T-cell lymphoma (except allogeneic SCT for hepatosplenic lymphoma), and ASCT in remitting relapsed/refractory aggressive B-cell lymphoma and (if first-line immunochemotherapy was < 2 years ago) for relapsed/refractory follicular and nodal marginal zone lymphoma. A PET/CT is done before every ASCT. Patients who have attained PET/CT-CR proceed to ASCT, having a good chance for cure in aggressive lymphoma (2-year PFS: relapsed/refractory aggressive B-cell lymphoma 75%, relapsed/refractory classical Hodgkin lymphoma 89%, peripheral T-cell lymphoma 78%). In the small number of patients with PET/CT-PR-local (a solitary active tumour), patients are given local irradiation after ASCT, with an acceptable cure rate (2-year PFS, 60%). Patients who do not attain PET/CT-CR or PET/CT-PR-local are considered for treatment with CAR-T cells or trials with bispecific antibodies, or, if not available, experimental salvage regimens to induce better remission status and then ASCT.

With ASCT, the real treatment for lymphoma is the preceding conditioning regimen. Several conditioning regimens exist. Between 2015 and 2019, we used BEAC as an alternative to BEAM because of melphalan shortages. The patients treated with BEAC showed inferior LSS, compared with those who received BEAM. It should be noted that Karolinska’s BEAM regimen, used throughout this 25-year period, has its own cytarabine schedule, with daily 400 mg/m^2^ 12-h infusions, instead of the more common [33] two daily 200 mg/m^2^ 1-h infusions. Infusion times correlate with cytarabine efficacy and toxicity, since the exposure to ara-C triphosphate (the active metabolite) is increased with longer infusions (cytarabine disappears from the plasma with a half-life of 7–13 min) [34, 35].

## Conclusion

We conclude that an increasingly PET/CT-based selection of patients prior to ASCT has improved outcome after ASCT for lymphoma. For patients with PET/CT-CR, conditioning with BEAM followed by ASCT remains a strong standard treatment for several lymphoma entities. Further research is needed to identify the place of ASCT among other emerging salvage techniques.

## Data Availability

The datasets during and/or analysed during the current study available from the corresponding author on reasonable request.

## Abbreviations

ASCT: Autologous stem-cell transplantation
CAR: Chimeric antigen receptor
CR: Complete response
CT: Computerized tomography
LSS: Lymphoma-specific survival
NRM: Non-relapse mortality
OS: Overall survival
PET/CT: Positron emission tomography/computerized tomography
PFS: Progression-free survival
PR: Partial response
TRM: Treatment-related mortality
